# Interactions between natural killer cells and dendritic cells favour T helper1-type responses to BCG in calves

**DOI:** 10.1186/s13567-016-0367-4

**Published:** 2016-08-17

**Authors:** Carly A. Hamilton, Suman Mahan, Gary Entrican, Jayne C. Hope

**Affiliations:** 1 The Roslin Institute, University of Edinburgh, Easter Bush, Midlothian, EH25 9RG Scotland, UK; 2 Moredun Research Institute, Pentlands Science Park, Bush Loan, Midlothian, EH26 0PZ Scotland, UK; 3 Zoetis, Portage Street, Kalamazoo, MI 49007 USA

## Abstract

Vaccination of neonatal calves with BCG induces a significant level of protection from infection with *Mycobacterium bovis*, the causative agent of bovine tuberculosis. Since neonatal vaccination of humans with BCG induces activation of NK cells, and young calves have high circulating numbers of these cells, we hypothesised that NK cells are important in the protective response to BCG. Furthermore, since NK cells play a role in shaping adaptive immune responses through interactions with DCs, we investigated the interactions between NK cells and DCs in the context of BCG. DCs infected with BCG expressed significantly higher levels of MHC class II and the co-stimulatory molecules CD40 and CD80, alongside augmented production of the Th1 polarising cytokine IL-12, when compared with uninfected DCs. Following in vitro co-culture with BCG-infected DCs, NK cells increased their expression of the activatory molecule CD25, with preferential activation of the CD2− NK cell subset. NK cell effector function, as measured by production of IFN-γ, was also significantly enhanced following co-culture with BCG-infected DCs. This study provides novel evidence to demonstrate that NK cells phenotypically and functionally mature after interactions with DCs in the context of BCG. Furthermore, through the production of IFN-γ and IL-12 by NK cells and DCs respectively, this interaction may drive protective Th1-type immune responses to *Mycobacteria*.

**Electronic supplementary material:**

The online version of this article (doi:10.1186/s13567-016-0367-4) contains supplementary material, which is available to authorized users.

## Introduction

Bovine tuberculosis (bTB), caused by infection of cattle with *Mycobacterium bovis* (*M. bovis*), is increasing in incidence in the United Kingdom (UK) and currently costs the economy up to £100 million per annum to control. Vaccination is an aspirational cornerstone for future bTB disease control, alongside improved diagnostic tests which differentiate infected from vaccinated animals (DIVA). However, currently there are no vaccines licensed for use in cattle. Despite over a century of research, Bacille Calmette Guerin (BCG) which is a live attenuated strain of *M. bovis*, remains the only vaccine available for use in humans against TB and is particularly effective when delivered to infants [1, 2]. Similarly, a number of studies have demonstrated that experimental vaccination of neonatal calves with BCG provides significant protection against *M. bovis* infection [3–5]. Although the use of BCG in the field is prohibited by EU legislation due to non-specific sensitisation to the tuberculin skin test, BCG remains the most effective vaccine available for use in cattle and the development of new DIVA tests may allow it to be deployed in the UK for bTB control. Alongside this, understanding the mechanisms by which BCG can induce protective immunity in young calves could pinpoint targets for improved vaccine design or delivery.

BCG vaccination of infant humans induces activation of innate effector cells such as natural killer (NK) cells and γδ T cells [6]. Since young calves, particularly those aged between 8 and 120 days old, have increased circulating numbers of NK cells [7–9], it was hypothesised that NK cells may play a role in the enhanced efficacy of BCG in neonatal calves. Similar to young calves, human infants have elevated levels of NK cells which also decline with age [10, 11], therefore research focusing on the role of NK cells during mycobacterial infection or vaccination in neonatal calves may also be applicable to studies in humans.

NK cells are large granular lymphocytes which were identified in the 1970s by their ability to lyse malignant or transformed cells without prior sensitisation [12]. This heterogeneous cell population has diverse roles in the immune system and are the first line of defence in the control of viruses, bacteria and parasites [13–16]. NKp46 is a natural cytotoxicity receptor (NCR) expressed exclusively by NK cells (NCR1; CD335) and commonly used as a pan-species marker to identify NK cells [17]. The development of a monoclonal antibody (mAb) specific to this NCR has facilitated the detailed study of NK cells in cattle [18]. Bovine NK cells lack expression of CD3 and can be subdivided into NKp46+ CD2+ and NKp46+ CD2low or CD2negative (referred to as CD2− herein) subsets [18]. These subsets of bovine NK cells differ in their localisation, phenotype and function. For example, the majority of peripheral blood derived NK cells are CD2+ and a small population are CD2−. In contrast, CD2− NK cells are the predominant subset found within lymph nodes and this subset has also been defined as the major NK cell subset present within skin draining afferent lymphatic vessels [18, 19]. CD2− NK cells have a higher expression of the activation markers CD25 and CD44, an increased proliferative capacity and enhanced ability to produce IFN-γ in comparison to their CD2+ counterparts. However, both subsets have equal cytotoxic capacities [20].

NK cells are traditionally regarded as cells of the innate immune system but can be viewed as an interface between innate and adaptive immunity due to their capacity to drive adaptive immune responses. Early interactions between populations of innate immune cells, particularly NK cells and dendritic cells (DCs), can influence the nature of the adaptive immune response. Protective immunity against *M. bovis* infection in cattle is driven by Th1-type immune responses which are characterised by IFN-γ production [21]. Initial investigations into bovine innate immune cell interactions in the context of mycobacteria showed that a population of NK-like cells from naïve calves produced IFN-γ after interplay with BCG-infected DCs [22]. More recently, interactions between NKp46+ CD2− NK cells and *M. bovis*-infected DCs were defined and showed that reciprocal interactions occur, resulting in activation of both CD2− NK cells and DCs [23]. Whether reciprocal interactions between NK cells and DCs occur in the context of BCG, and the key mechanisms involved, has not been elucidated in cattle. Furthermore, the effect of these interactions on the induction of the adaptive immune response is unknown. To study the interaction between these two populations of innate immune cells, in vitro co-cultures between BCG-infected monocyte-derived DCs and autologous NK cells enriched from peripheral blood were established. We hypothesised that NK cells required interactions with BCG-infected DCs to become phenotypically and functionally mature and the interaction between the NK cells and DCs drives Th1-type immune responses. To address this hypothesis we developed in vitro co-cultures between BCG-infected monocyte-derived DCs and autologous NK cells enriched from peripheral blood and investigated the phenotypic and functional characteristics of the resulting populations. We demonstrated that monocyte-derived DCs increased their expression of MHC class II, CD40 and CD80 in response to exposure to BCG in conjunction with enhanced production of the Th1 polarising cytokine IL-12. As a result of co-culture with BCG-infected DCs, NK cells became activated and produced increased amounts of IFN-γ, indicating that this interaction may drive Th1 immune responses through the production of IL-12 and IFN-γ by DCs and NK cells respectively.

## Materials and methods

### Experimental animals

Experiments were performed using male British Holstein–Friesian (*Bos taurus*) calves obtained from the Langhill herd, University of Edinburgh which has been certified free of bTB for over 10 years. All calves were housed at Dryden Farm, University of Edinburgh according to Home Office guidelines and with approval from The Roslin Institute Local Ethics Committee. Calves were under 6 months of age.

### Isolation of peripheral blood mononuclear cells (PBMCs)

Blood was collected by jugular venepuncture into sodium heparin (Fisher Scientific, UK) and PBMCs were separated by density gradient centrifugation using Histopaque 1083 (Sigma-Aldrich, UK).

### Isolation of CD14+ monocytes and culture of monocyte-derived DCs

CD14+ monocytes were positively selected from PBMCs using MACS anti-human CD14 MicroBeads and a MidiMACS LS column (Miltenyi Biotec, UK) to purities >98% (Additional file 1A). Monocytes were cultured in RPMI 1640 (Gibco, UK), 10% foetal calf serum (TCS Biosciences Ltd, UK), 5 × 10^−5^M 2-mercaptoethanol (Sigma Aldrich, UK) and 50 µg/mL gentamicin (Sigma-Aldrich, UK) supplemented with recombinant bovine GM-CSF and IL-4 (generated at Moredun Research Institute, UK) for 3 days to obtain immature DCs as previously described [24]. DCs were harvested by incubating with non-enzymatic cell dissociation fluid (Sigma-Aldrich, UK). Floating and adherent cells were pooled together and live DCs were identified by gating as FSC^high^ SSC^high^ cells (Additional file 1B) which were negative for the dead cell discriminator Sytox Blue (Life Technologies, UK) (Additional file 1C).

### Infection of monocyte-derived DCs with BCG or BCG-fluorescein isothiocyanate (FITC)

BCG Vaccine Danish strain 1331 (Statens Serum Institut, Denmark) was prepared by reconstituting lyophilised BCG with 1 mL of diluted Sauton medium (Statens Serum Institut, Denmark). For fluorescent labelling of BCG, BCG was mixed with an equal volume of 0.2 mg/mL FITC (Sigma Aldrich, UK) for 3 h and washed 3 times prior to infection of monocyte-derived DCs. DCs were infected with BCG or BCG-FITC at a multiplicity of infection (MOI) of 5 for 42 h.

### Isolation of NK cells

NKp46+ NK cells were positively selected from peripheral blood by labelling PBMCs with mouse anti-ovine CD335 (EC1.1, IgG1, kindly provided by Dr Timothy Connelley) and immunomagnetic anti-mouse pan IgG Dynabeads (Invitrogen, UK) to purities >95% (Additional file 2). NK cells were cultured in RPMI 1640 (Gibco, UK) supplemented with 10% foetal calf serum (TCS Biosciences Ltd, UK), 5 × 10^−5^M 2-mercaptoethanol (Sigma-Aldrich, UK) and 50 µg/mL gentamicin (Sigma Aldrich, UK). Magnetic beads were removed after 24 h of culture using a Dyna-Mag 15 magnet (Invitrogen, UK).

### Co-culture of NK cells with BCG-infected DCs

BCG-infected DCs or uninfected DCs were co-cultured for 18 h with autologous NK cells at a previously determined optimal NK cell: DC ratio of 5:1 [23]. Following co-culture, cells were pelleted and supernatants were collected and stored at −20 °C. NK cells cultured alone, with BCG (MOI of 5) or supplemented with recombinant bovine IL-12 (20 BU/mL) and recombinant human IL-18 (20 ng/mL) served as control conditions.

### Multi-colour immunofluorescent staining

NK cells were identified by labelling cells with mouse anti-bovine CD335-PE (AKS1, IgG1, Bio-Rad AbD Serotec, UK) and mouse anti-bovine CD3 (MM1A, IgG1, VMRD, Washington) indirectly conjugated to goat anti-mouse IgG1-AF647 (Life Technologies, UK). Subsets of NK cells were differentiated by labelling with mouse anti-bovine CD2-FITC (CC42, IgG1, Bio-Rad AbD Serotec). Expression of CD25 was assessed by labelling NK cells with mouse anti-bovine CD25 (CACT108A, IgG2a, VMRD, Washington) and goat anti-mouse IgG2a-AF647 (Life Technologies, UK). DCs were identified as described in Additional file 1. Mouse anti-bovine MHC class II-PE (CC158, IgG2a, Bio-Rad AbD Serotec), mouse anti-bovine CD40-FITC (IL-A156, IgG1, Bio-Rad AbD Serotec) and mouse anti-bovine CD80 (IL-A159, Bio-Rad AbD Serotec) indirectly conjugated to goat anti-mouse IgG1-AF647 (Life Technologies, UK) were used to determine DC expression of MHC class II, CD40 and CD80 respectively. Live cells were identified using Sytox Blue (Life Technologies, UK) and 50 000 events were collected using the BD LSRFortessa and FACSDiva Software. Gates were established by using Fluorescence Minus One (FMO) controls. Data analysis was performed using FlowJo v10 software (Treestar Inc, USA).

### Enzyme linked immunosorbent assay (ELISA)

ELISAs were performed to detect the presence of bovine IL-12 [25] and IFN-γ [26] as previously described. Absorbance was measured at 450 nm subtracted from 690 nm using the Synergy HT Multi-Mode Microplate Reader and Gen 5 software.

### Data analysis

Data analysis was performed using Microsoft Excel 2010 and GraphPad Prism 6. Statistical analysis was completed using Minitab v16. Distribution of data was assessed using a normality test with *p* > 0.05 deemed to be normally distributed data. Statistical methods used are detailed in individual figure legends. *p* < 0.05*, *p* < 0.01** and *p* < 0.001***.

## Results

### Infection of bovine monocyte-derived DCs with BCG results in increased expression of MHC class II, CD40, CD80 and production of the Th1 polarising cytokine IL-12

**Figure 1:**
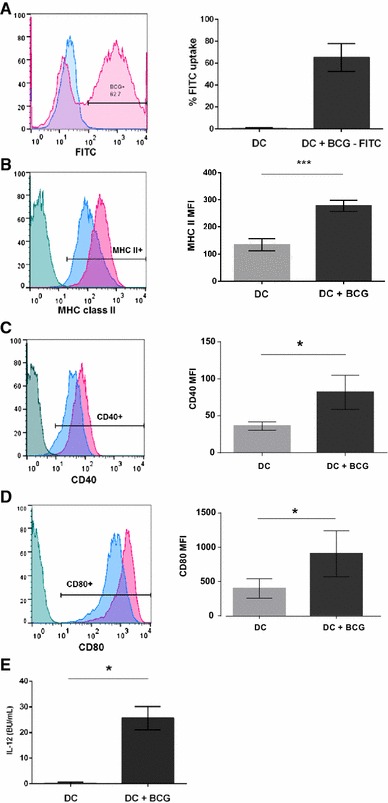
**BCG is taken up by bovine monocyte-derived DCs resulting in increased the expression of MHC class II, CD40 and CD80 and production of the Th1 polarising cytokine IL-12.** Monocyte-derived DCs were cultured for 3 days and infected with FITC-labelled BCG (MOI 5) for 42 h. FACS plots from one representative animal (**A**) illustrate the percentage uptake of BCG-FITC by uninfected DCs (blue histogram) and BCG-infected DCs (pink histogram). Gates were set using uninfected DCs. Pooled data from four calves (**A**) illustrate the average percentage uptake of BCG-FITC ± SD by uninfected DCs (lighter bar) and BCG-infected DCs (darker bar). Uninfected and BCG-infected DCs were labelled with mAbs for MHC class II, CD40 and CD80 and analysed by flow cytometry. FACS plots from one representative animal show the expression of MHC class II (**B**), CD40 (**C**) and CD80 (**D**) by uninfected (blue histograms) and BCG-infected DCs (pink histograms). Positive cells were identified based on FMO controls (green histograms). Pooled data from four calves indicates the average MFI ± SD of MHC class II (**B**), CD40 (**C**) and CD80 (**D**) expression by uninfected (lighter bars) and BCG-infected DCs (darker bars). Supernatants were retrieved from uninfected and BCG-infected DCs and assayed for IL-12 production by ELISA. Pooled data from five calves represent the average levels ± SD of IL-12 BU/mL produced by uninfected (lighter bars) and BCG-infected DCs (darker bars) (**E**). Data were normally distributed (*p* > 0.05) and significance between uninfected and BCG-infected DCs was assessed using a 2-sample *t* test. *p* < 0.05*, *p* < 0.001***.

To assess the percentage of monocyte-derived DCs infected by BCG, DCs were exposed to fluorescently labelled BCG at an MOI of 5. BCG infected a significant proportion of immature DCs with 64.5% (48.2–75.8%; SD = 14.1) shown to contain BCG-FITC (Figure 1A). The effect of BCG on the phenotype of DCs was investigated by comparing the expression of MHC class II (Figure 1B) and the co-stimulatory molecules CD40 (Figure 1C) and CD80 (Figure 1D) by uninfected and BCG-infected DCs. Expression of MHC class II (*p* < 0.001), CD40 (*p* = 0.032) and CD80 (*p* = 0.044) were significantly enhanced following infection of DCs with BCG compared with uninfected DCs. Uninfected DCs expressed MHC class I and CD86, however no significant increases were observed in the expression of these molecules by BCG-infected DCs (data not shown). Since protective immunity against *M. bovis* infection in cattle is driven by Th1 polarised immune responses [21], production of the Th1 polarising cytokine IL-12 by uninfected and BCG-infected DCs was measured. DCs infected with BCG secreted significantly higher levels of IL-12 (*p* = 0.023) compared with uninfected DCs (Figure 1E).

### CD25 expression by bovine CD2− NK cells was significantly increased following co-culture with autologous BCG-infected DCs

**Figure 2:**
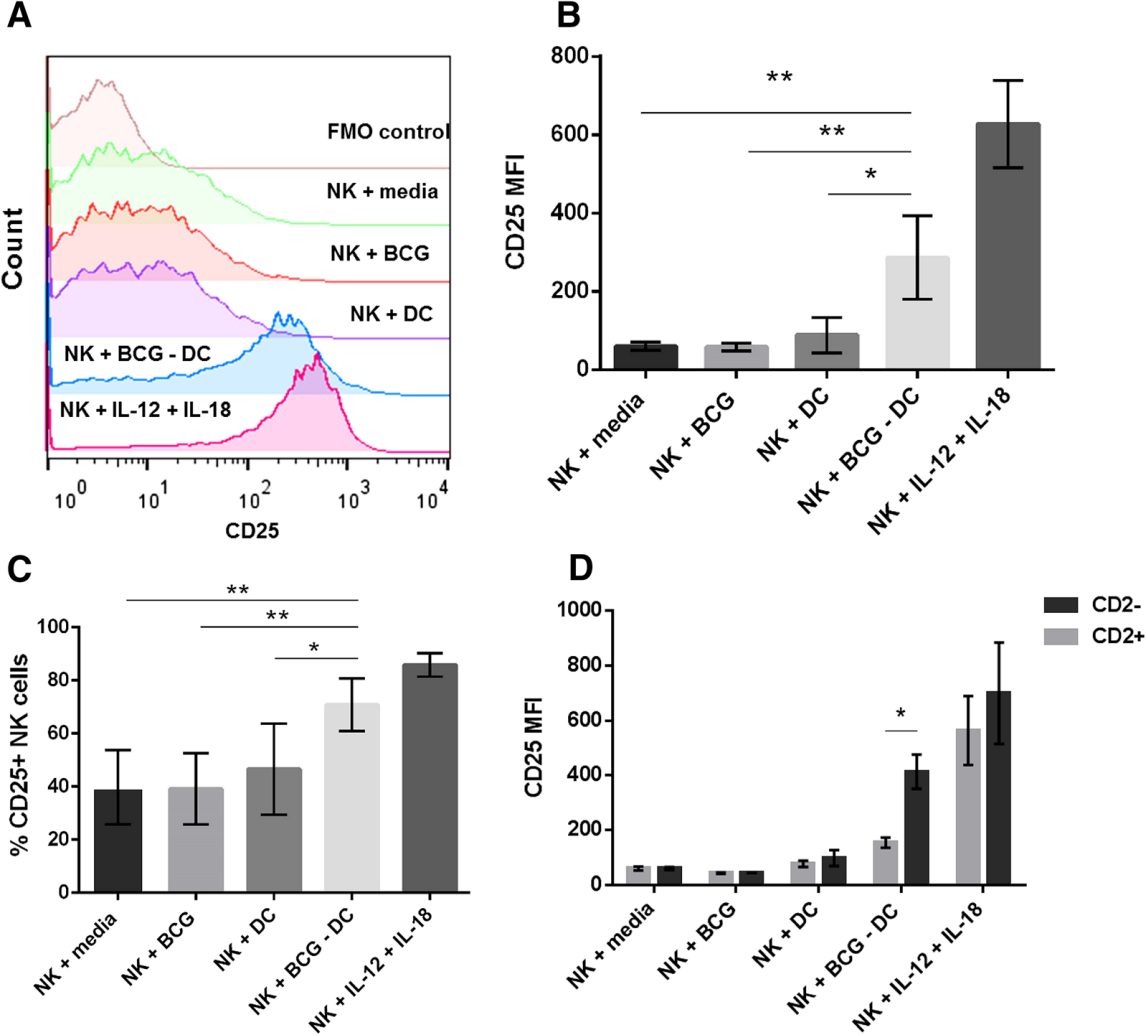
**CD25 expression by bovine CD2− NK cells was significantly increased following co-culture with autologous BCG-infected DCs.** Monocyte-derived DCs were cultured for 3 days and infected with BCG (MOI 5) for 24 h. Autologous NKp46+ cells were enriched from peripheral blood and cultured with BCG-infected DCs at a ratio of 5 NK cells per DC. NK cells cultured with media, BCG, uninfected DCs or with recombinant bovine IL-12 and recombinant human IL-18 served as controls. After 18 h of co-culture, cells were labelled with mAbs for NKp46, CD2 and CD25 and analysed by flow cytometry. FACS plots from one representative animal illustrate the expression of CD25 by NKp46+ NK cells after culture with media, BCG, uninfected DCs, BCG-infected DCs or with IL-12 and IL-18 (**A**). Positive cells were identified based on FMO controls. Pooled data from five calves illustrate the average MFI ± SD of CD25 expression by NK cells (**B**). Pooled data from five calves displays the average percentage of CD25+ NK cells ± SD (**C**). Pooled data from five calves indicate the average MFI ± SD of CD25 expression by CD2+ (lighter bars) and CD2− (darker bars) NK cells (**D**). Data were normally distributed (*p* > 0.05) and significance between co-culture conditions was assessed using 2-sample t tests. *p* < 0.05*, *p* < 0.01**.

In vitro co-cultures between BCG-infected DCs and autologous NK cells were established to assess the interaction between NK cells and DCs in the context of BCG. CD25 was used to assess the activation status of NK cells after in vitro co-culture with BCG-infected DCs. CD25 expression, as represented by the mean fluorescence intensity (MFI), was low when NK cells were cultured with media, with BCG and with uninfected DCs (Figure 2B). During co-culture with BCG-infected DCs, NK cell expression of CD25 was significantly increased compared with NK cells cultured with media (*p* = 0.009), with BCG (*p* = 0.009) or with uninfected DCs (*p* = 0.012). NK cells supplemented with IL-12 and IL-18 (positive control), had a high expression of CD25 (Figure 2B). In addition to enhanced CD25 expression (as assessed by MFI) a similar significant increase in the percentage of CD25+ NK cells present after culture with BCG-infected DCs was evident compared with the percentage of CD25+ NK cells present after culture of NK cells with media (*p* = 0.005), with BCG (*p* = 0.004) and with uninfected DCs (*p* = 0.034) (Figure 2C). To determine if the observed activation of NK cells in response to co-culture with BCG-infected DCs (Figures 2A–C) was attributed to a particular subset of bovine NK cells, the expression of CD25 by CD2+ and CD2− subsets of NK cells was assessed. Expression was compared following culture of NK cells with media, BCG, uninfected DCs, BCG-infected DCs or when stimulated with IL-12 and IL-18. CD25 expression did not significantly differ between CD2+ and CD2− subsets when NK cells were cultured with media, BCG, uninfected DCs or with IL-12 and IL-18. However, after culture with BCG-infected DCs, CD2− NK cells had a significantly higher expression of CD25 than CD2+ NK cells (*p* = 0.017) reflecting preferential activation of CD2− NK cells (Figure 2D).

A population of NKp46+ CD3+ lymphocytes which have shared attributes of both bovine NK cells and T cells has recently been described [27]. As the enrichment technique used herein to isolate bovine NK cells from peripheral blood involves positively selecting the total NKp46+ population, the observed activation of NK cells as a result of in vitro co-culture with BCG-infected DCs (Figures 2A–C) could be due (at least in part) to activation of these double positive cells and not solely to NKp46+ CD3- NK cells. To address whether NKp46+ CD3+ cells were activated after co-culture with BCG-infected DCs, the expression of CD25 was analysed on NKp46+ CD3+ and NKp46+ CD3− cells. CD3− cells accounted for a significantly higher (*p* < 0.001) percentage of the NKp46+ CD25+ population in comparison to CD3+ cells, with 98.4% (97.7–98.9%; SD = 0.51) of the NKp46+ CD25+ population being CD3− (Additional file 3).

### Production of IFN-γ by bovine NK cells was significantly increased following co-culture with autologous BCG-infected DCs

**Figure 3:**
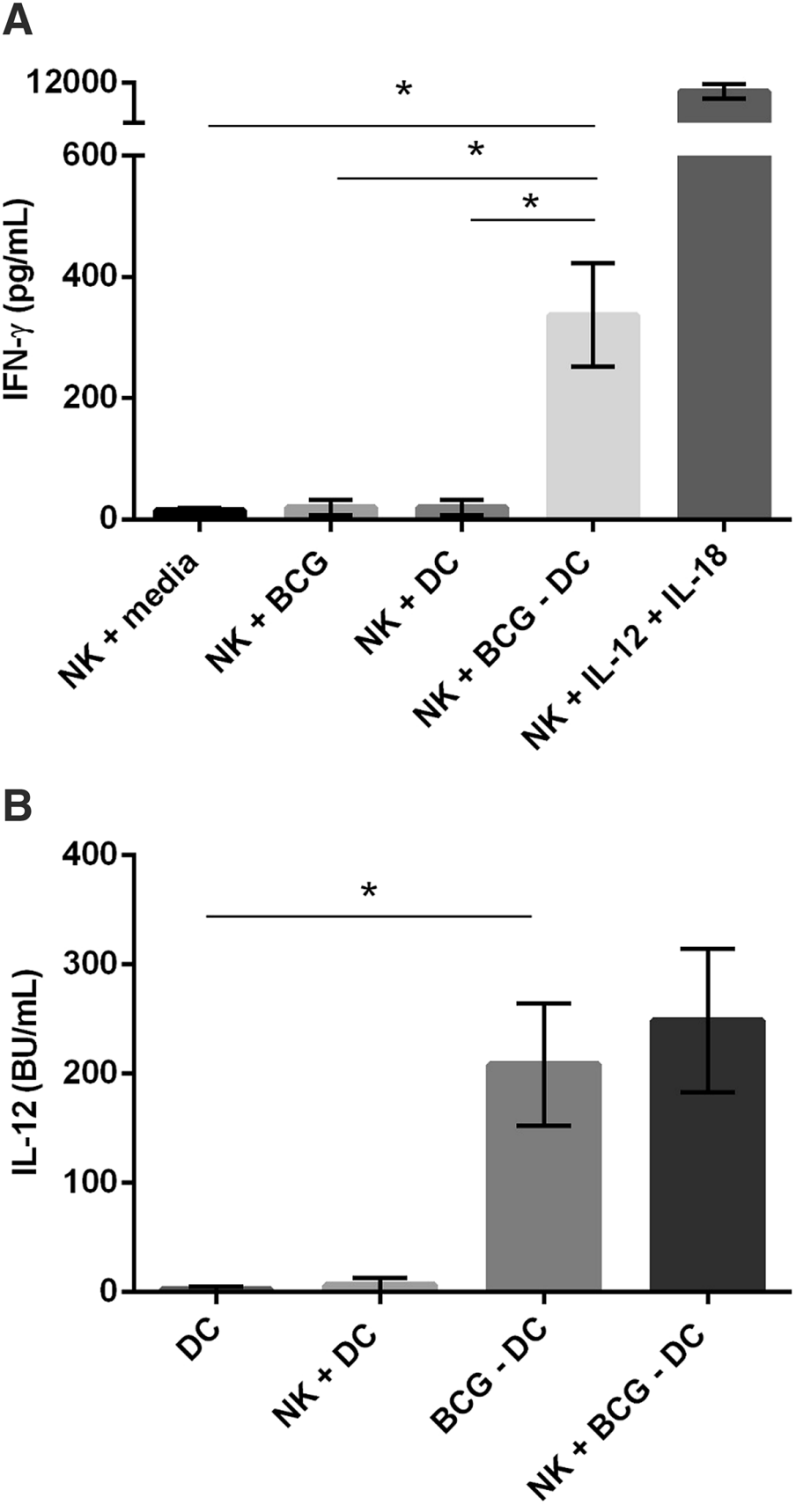
**IFN-γ production by NK cells was significantly increased following co-culture with autologous BCG-infected DCs.** Monocyte-derived DCs were cultured for three days and infected with BCG (MOI 5) for 24 h. Autologous NKp46+ cells were enriched from peripheral blood and cultured with BCG-infected DCs at a ratio of 5 NK cells per DC. NK cells cultured with media, BCG, uninfected DCs or with recombinant bovine IL-12 and recombinant human IL-18 served as controls. After 18 h of co-culture, supernatants were assayed for IFN-γ and IL-12 production by ELISA. Pooled data from five calves represents the average levels ± SD of IFN-γ (pg/mL) produced (**A**). Pooled data from five calves represents the average levels ± SD of IL-12 (BU/mL) produced (**B**). Data were normally distributed (*p* > 0.05) and significance between co-culture conditions was assessed using 2-sample t tests; *p* < 0.05*.

In addition to the activation status of NK cells, the effector function of NK cells following co-culture with BCG-infected DCs was investigated by determining production of IFN-γ. Supernatants were retrieved from co-culture experiments and assayed for IFN-γ by ELISA. IFN-γ production was low when NK cells were cultured with media, with BCG or with uninfected DCs. Following co-culture with BCG-infected DCs, NK cell production of IFN-γ was significantly augmented compared with NK cells cultured with media (*p* = 0.020), NK cells cultured with BCG (*p* = 0.021) and NK cells cultured with uninfected DCs (*p* = 0.021). NK cells supplemented with IL-12 and IL-18 (positive control) produced high levels of IFN-γ (Figure 3A). Supernatants were retrieved from co-culture experiments and assayed for the presence of IL-12 by ELISA. As illustrated previously (Figure 1E), DCs secreted elevated levels of IL-12 upon infection with BCG compared with the very low levels produced by uninfected DCs. Similar to DCs cultured with media alone, uninfected DCs cultured with NK cells produced low levels of IL-12. Addition of NK cells to BCG-infected DCs did not significantly increase the level of IL-12 produced by BCG-infected DCs alone (Figure 3B).

## Discussion

To determine the effect of BCG on interactions between NK cells and monocyte-derived DCs, in vitro co-cultures between these two populations of innate immune cell populations were established. Prior to assessment of the effect of co-culture with BCG-infected DCs on the activation and effector function of NK cells, the influence of the vaccine strain BCG Danish on DC phenotype and cytokine production was determined. CD14+ monocytes cultured with recombinant bovine GM-CSF and IL-4 for 3 days represent a population of immature monocyte-derived DCs [24]. Immature monocyte-derived DCs ingested BCG as illustrated by the uptake of fluorescently labelled bacteria (Figure 1A). Receptors involved in the uptake of mycobacteria by human and murine DCs include dendritic cell-specific ICAM-3-grabbing nonintegrin (DC-SIGN) [28], Toll-like receptor (TLR) 2 [29], TLR4 [30] and TLR9 [31]. Similarly, a role for DC-SIGN in the uptake of BCG by bovine monocyte-derived DCs was confirmed when it was demonstrated that blocking DC-SIGN with a polyclonal antibody reduced binding of green fluorescent protein (GFP)-labelled BCG [32]. Upon antigen uptake, immature DCs undergo maturation characterised by the upregulation of MHC class I, MHC class II and costimulatory molecules, including CD40, CD80 and CD86 [33]. Following uptake of BCG by bovine monocyte-derived DCs, DCs significantly increased their expression of MHC class II (Figure 1B), CD40 (Figure 1C) and CD80 (Figure 1D) reflecting maturation of DCs in response to BCG. This is in line with published data whereby DCs infected with BCG Pasteur showed an increased expression of MHC class II, CD40 and CD80 [34]. Taken together, this provides evidence that bovine monocyte-derived DCs undergo maturation in response to BCG Pasteur and to the human vaccine strain BCG Danish. In parallel with phenotypic maturation in response to antigen uptake, DCs also secrete cytokines which direct adaptive immune responses, and therefore link the innate and the adaptive immune response. Naïve CD4+ T cells can differentiate into various subsets of CD4+ T helper cells which is dictated in part by the cytokine milieu present at the time of differentiation. For example, the presence of IL-12 and IFN-γ in the local environment results in the development of a Th1 immune response whereas the presence of IL-4 directs a Th2 immune response [35]. Given that a Th1 dominant response is likely to be most effective against *M. bovis* infection, the production of IL-12 by uninfected and BCG-infected DCs was quantified. BCG-infected DCs produced significant levels of IL-12 after infection with BCG indicating that BCG-infected DCs could contribute significantly to the induction of a CD4+ Th1 immune response. Bovine DCs have been shown previously to secrete IL-12 after infection with *M. bovis* and the Pasteur strain of BCG [34]. The results presented in Figure 1E demonstrate that DCs can also produce IL-12 when stimulated with the vaccine strain of BCG.

After establishing that DCs undergo maturation in response to infection with BCG (Figures 1B–D) and produced elevated levels of the Th1 polarising cytokine IL-12 (Figure 1E), the effect of BCG-infected DCs on NK cell activation was investigated by assessing NK cell expression of CD25. CD25 expression was significantly augmented when NK cells were cultured with BCG-infected DCs, reflecting activation of NK cells in response to co-culture with DCs in the context of BCG (Figures 2A–C). CD25 is the α chain of the IL-2R and together with the IL-2Rβ and γ chains allows IL-2 signalling through the IL-2R, therefore data presented in Figures 2A–C suggests NK cells are more responsive to IL-2 following co-culture with BCG-infected DCs. The observed activation of NK cells after in vitro co-culture with BCG-infected DCs was due to preferential activation of the CD2− subset of NK cells illustrated by a significantly higher CD25 expression by CD2− NK cells compared with CD2+ NK cells (Figure 2D). Interestingly, in the positive control whereby NK cells were stimulated with IL-12 and IL-18, there was not a significant difference between the expression of CD25 by the two subsets, indicating that the increased activation of CD2− NK cells after co-culture with BCG-infected DCs was unique to these conditions. This preferential activation of bovine CD2− NK cells was also apparent when NK cells were cultured with *M. bovis*-infected DCs [23] showing that CD2− NK cells are favourably activated when NK cells are cultured with DCs in vitro in the context of both BCG and *M. bovis*. In line with this finding, CD2− NK cells are the main subset of NK cells migrating in bovine afferent lymph [19], they preferentially migrate towards *M. bovis*-infected DCs during in vitro chemotaxis assays [23] and are key producers of IFN-γ [20]. Therefore, we hypothesise that CD2− NK cells migrate to lymph nodes in vivo where they interact with DCs, resulting in NK cell activation. It was also demonstrated in Additional file 3 that activation of NKp46+ cells after co-culture with BCG-infected DCs was specific to NKp46+ CD3− NK cells and that a recently characterised subset of lymphocytes that co express NKp46 and CD3 were not activated by co-culture with BCG-infected DCs [27].

Production of immunoregulatory cytokines, primarily IFN-γ, is a key functional property of NK cells and in parallel with IL-12, early production of IFN-γ drives Th1 polarised immune responses. NK cell production of IFN-γ was significantly augmented when NK cells were co-cultured with BCG-infected DCs (Figure 3A), demonstrating that production of IFN-γ by NK cells after co-culture with BCG-infected DCs has the potential to prime Th1-type immunity. Murine NK cells polarise Th1 immune responses through interactions with DCs [36] and enhanced secretion of IFN-γ by NK cells was also noted after reciprocal interactions with *M. tb*-infected DCs [37]. Within the present study, the subset of NK cells responsible for the increased IFN-γ production following co-culture with BCG-infected DCs was not defined. However, bovine CD2− NK cells have an increased capacity to produce IFN-γ compared with CD2+ NK cells [20]. Furthermore, CD2− NK cells are the subset responsible for production of IFN-γ after culture with *M. bovis*-infected DCs [23]. Therefore, we propose that CD2− NK cells are the major IFN-γ producers following co-culture with BCG-infected DCs. Studies in humans have shown that BCG can bind directly to NK cells through NK cell expression of TLR2 and NKp44 and induce NK cell production of IFN-γ [38–40]. Nevertheless, BCG alone had no effect on the production of IFN-γ by NK cells (Figure 3A), or on the activation of NK cells (Figures 2A–C). Despite TLR2 being transcribed by bovine NK cells (Nazneen Siddiqui, PhD thesis, Imperial College, 2011), expression of TLR2 was not detected on the surface of NK cells (data not shown). Furthermore, NKp44 is a pseudogene in cattle [41] therefore combined with the lack of surface TLR2, may explain why there was no direct recognition of BCG by bovine NK cells in this study.

Data presented herein demonstrates that BCG-infected DCs provide signals to NK cells which induce phenotypical and functional changes, however NK cells are also thought to modulate the response of DCs in a reciprocal interaction. Thus, the effect of NK cells on the production of IL-12 by BCG-infected DCs was assessed. The addition of NK cells increased the amount of IL-12 produced by BCG-infected DCs alone; although this was not significant (Figure 3B). NK cells express receptors for IL-12 [42] and as the supernatants were retrieved 18 h after co-culture with BCG-infected DCs, it is possible that the presence of NK cells enhanced the production of IL-12 by BCG-infected DCs through the release of IFN-γ, and this IL-12 was subsequently utilised by NK cells expressing IL-12R. In mice, DCs that have been in contact with NK cells were shown to increase secretion of IL-12 [43] and in cattle, *M. bovis*-infected DCs showed enhanced expression of MHC class II after interactions with autologous peripheral blood NK cells [23]. Hence, NK cells may influence the adaptive immune response by inducing effective antigen presenting cells (APC). The production of Th1 polarising cytokines IFN-γ and IL-12 during the co-culture between NK cells and BCG-infected DCs suggests that this interaction may contribute to the induction of Th1 immune responses.

The mechanisms underlying the interactions between bovine NK cells and DCs in the context of BCG have not yet been elucidated. Both soluble factors and contact-dependent receptor ligand interactions are thought to be important. Since uninfected DCs (which have a low expression of MHC class II, CD40 and CD80, and very low levels of IL-12) did not stimulate NK cell activation (Figures 2A–C) or production of NK cell derived IFN-γ (Figure 3A), we hypothesise that IL-12, CD40 and CD80 are likely to be key molecules involved in the cross-talk between NK cells and DCs.

To conclude, in this study bovine NK cells required interactions with BCG-infected DCs for optimal activation and production of IFN-γ in vitro. Furthermore, CD2− NK cells preferentially interacted with DCs in the context of BCG highlighted by their increased activation compared with their CD2+ counterparts. CD2− NK cells are abundant within bovine afferent lymph [19] and lymph nodes [44] and therefore may traffic to lymph nodes and interact with DCs in vivo. Through the secretion of IFN-γ and IL-12 by NK cells and BCG-infected DCs respectively, this interaction generated a cytokine milieu favourable to polarisation of Th1 immune responses which are thought to be important in the protective immune response against *M. bovis* infection of cattle. The interaction between bovine CD2− NK cells and DCs may represent a target for future novel vaccination strategies to initiate Th1 polarised immune responses during BCG vaccination of neonatal calves.

## Additional files

10.1186/s13567-016-0367-4 **Culture of bovine monocyte-derived DCs.** CD14+ monocytes were positively selected using MACS MicroBeads conjugated to mouse anti-human CD14 antibody and labelled with goat anti-mouse IgG PE antibody to assess purity. A representative FACS plots indicates the purity of CD14+ monocytes from one animal with the pink histogram representing cells positive for CD14 (A). Gates were set using unstained cells (blue histogram). Purities of CD14+ monocytes were consistently >98%. CD14+ monocytes were cultured for three days with recombinant bovine GM-CSF and IL-4 to obtain monocyte-derived DCs. DCs were identified by gating FSC^high^ SSC^high^ cells (B) which were negative for the live cell discriminator, Sytox Blue (C).
10.1186/s13567-016-0367-4 **Isolation of bovine peripheral blood NK cells.** NK cells were isolated by labelling PBMCs with mouse anti-ovine NKp46 followed by positive selection using pan-mouse IgG Dynabeads. NK cell purity was assessed by labelling cells with mouse anti-ovine NKp46 indirectly conjugated to goat anti-mouse IgG PE. Representative FACS plots illustrate the gating strategy used to identify bovine NK cells. Single cells were gated (A), followed by lymphocytes (B) which were negative for the live cell discriminator Sytox Blue (C). Additional file 2D indicates the purity of NKp46+ NK cells (pink histogram). Gates were set using unstained cells (blue histogram). Purities of positively selected NK cells were consistently >95%.
10.1186/s13567-016-0367-4 **NKp46+ CD3+ lymphocytes were not activated following co-culture with BCG-infected DCs.** Monocyte-derived DCs were cultured for three days and infected with BCG for 24 h. NKp46+ cells were enriched from peripheral blood and cultured with infected DCs at a ratio of 5 NK cells per DC. After 18 h of co-culture, cells were labelled with mAbs for CD3, NKp46 and CD25 and analysed by flow cytometry. FACS plots from one representative animal denotes the gating strategy used to assess the percentage of CD3+ and CD3- cells within the total gated NKp46+ CD25+ cells. Gates were set using FMO controls. NKp46+ NK cells were selected (A) followed by CD25+ NK cells (B) and then CD3+ and CD3- cells were gated (C). Pooled data from three calves illustrates the percentage of CD3+ (lighter bar) and CD3- (darker bar) cells ± SD within the NKp46+ CD25 population (D). Data were normally distributed (*p* > 0.05) and significance between CD3+ and CD3- cells was assessed using a 2-sample t-test; *p* < 0.001***.

## Abbreviations

APC: antigen presenting cell
BCG: Bacille Calmette Guerin
BSA: bovine serum albumin
bTB: bovine tuberculosis
BU: biological unit
CD: cluster of differentiation
DCs: dendritic cells
DC-SIGN: dendritic cell-specific ICAM-3 grabbing non integrin
DIVA: differentiating infected from vaccinated animals
ELISA: enzyme-linked immunosorbent assay
EU: European Union
FBS: fetal bovine serum
FITC: fluorescein isothiocyanate
FMO: fluorescence minus one
FSC: forward scatter
GFP: green fluorescent protein
GM-CSF: granulocyte-macrophage colony stimulating factor
IFN: interferon
IL: interleukin
mAb: monoclonal antibody
*M. bovis*: *Mycobacterium bovis*
MFI: mean fluorescence intensity
MHC: major histocompatibility complex
MOI: multiplicity of infection
NCR: natural cytotoxicity receptor
PBMCs: peripheral blood mononuclear cells
PBS: phosphate-buffered saline
SSC: side scatter
Th1: T helper 1
TLR: toll-like receptor
