# Supplementary figures and images for: Interactions between natural killer cells and dendritic cells favour T helper1-type responses to BCG in calves
Source: Vet Res. 2016 Aug 17;47:85. doi: 10.1186/s13567-016-0367-4 (PMC4988014; doi:10.1186/s13567-016-0367-4)

## Slide 1
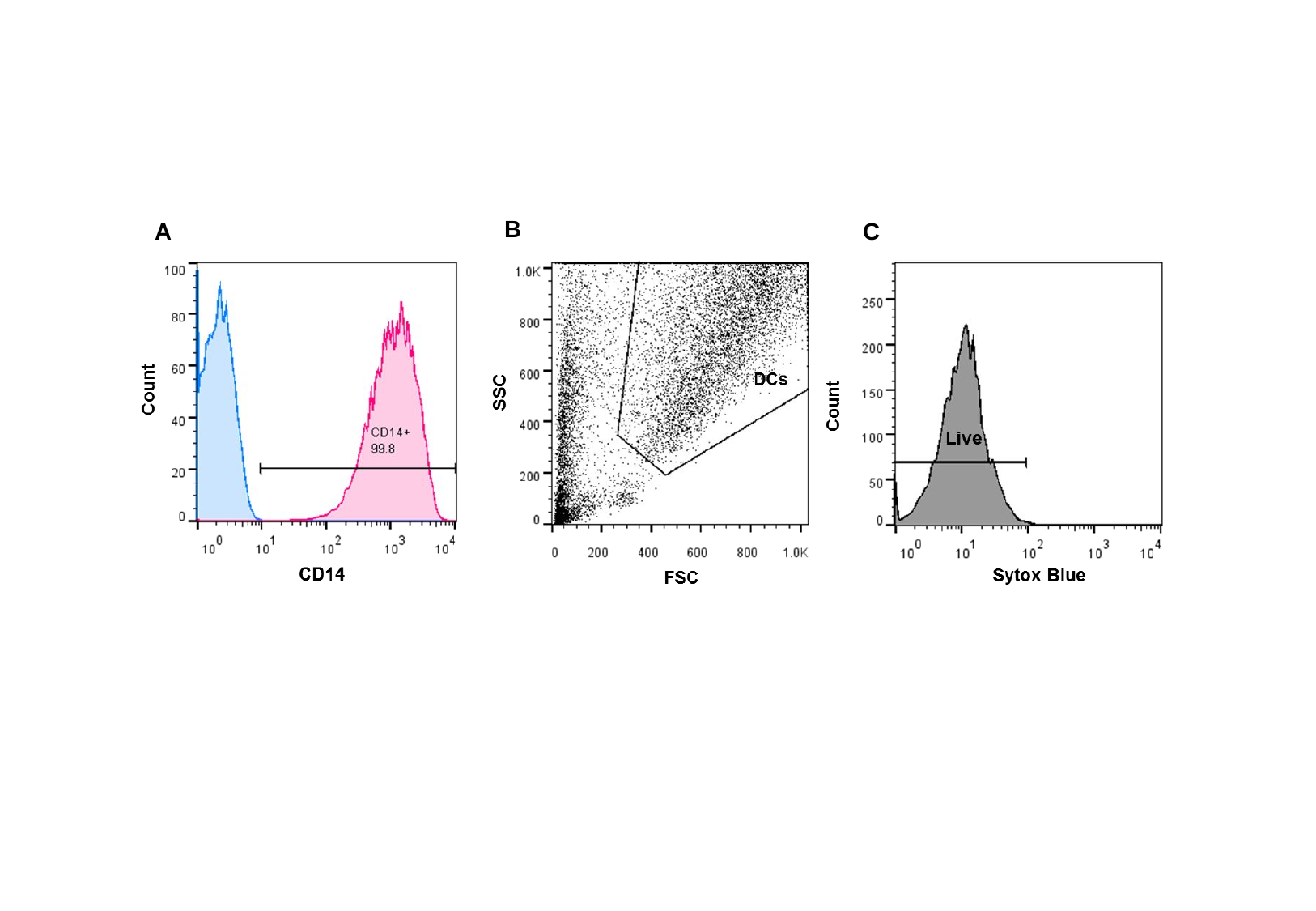

B
C
A

Supplement: Supplementary file 1 — 10.1186/s13567-016-0367-4 Culture of bovine monocyte-derived DCs. CD14+ monocytes were positively selected using MACS MicroBeads conjugated to mouse anti-human CD14 antibody and labelled with goat anti-mouse IgG PE antibody to assess purity. A representative FACS plots indicates the purity of CD14+ monocytes from one animal with the pink histogram representing cells positive for CD14 (A). Gates were set using unstained cells (blue histogram). Purities of CD14+ monocytes were consistently >98%. CD14+ monocytes were cultured for three days with recombinant bovine GM-CSF and IL-4 to obtain monocyte-derived DCs. DCs were identified by gating FSChigh SSChigh cells (B) which were negative for the live cell discriminator, Sytox Blue (C). [file 13567_2016_367_MOESM1_ESM.pptx]

## Slide 1
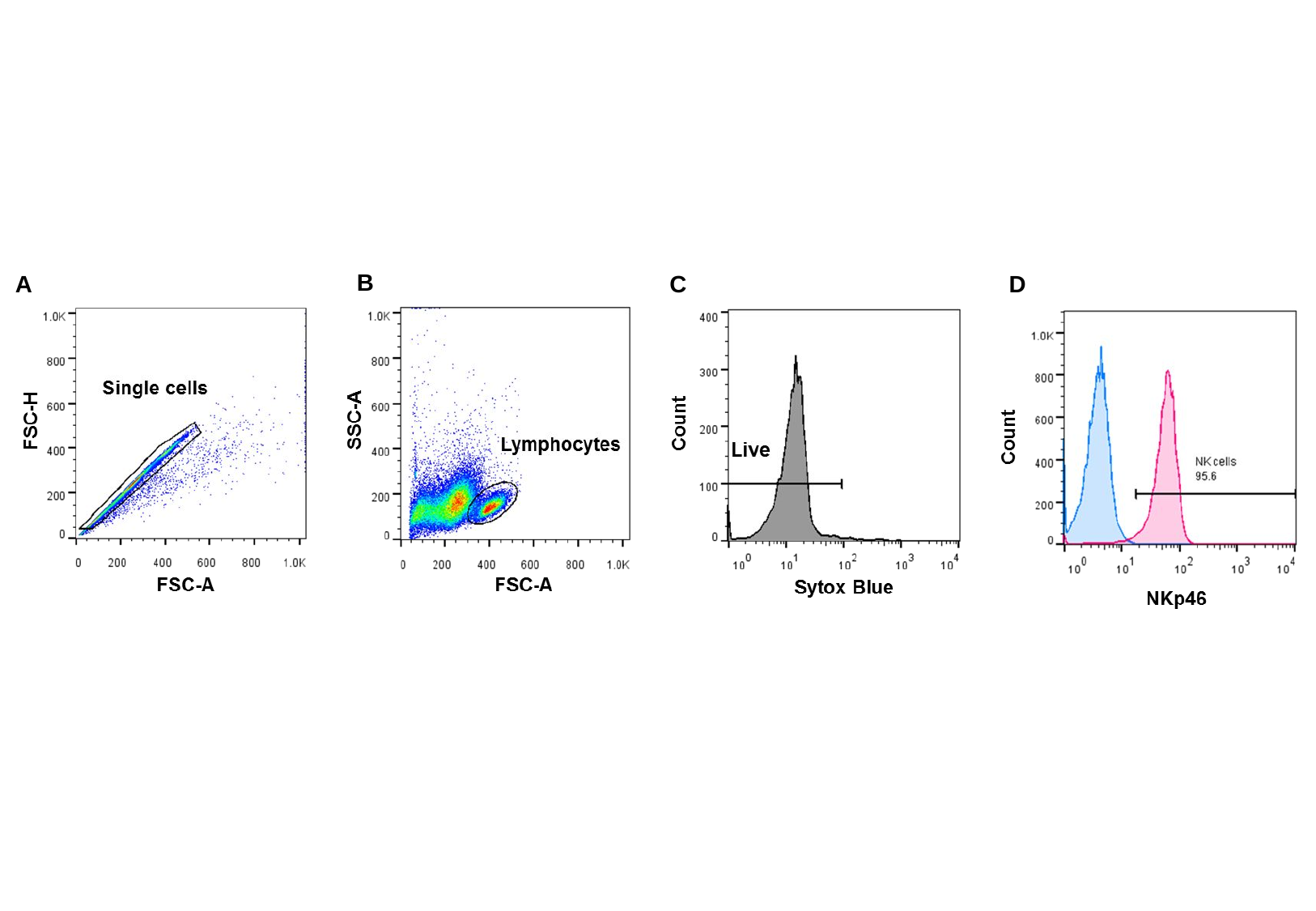

B
A
C
D

Supplement: Supplementary file 2 — 10.1186/s13567-016-0367-4 Isolation of bovine peripheral blood NK cells. NK cells were isolated by labelling PBMCs with mouse anti-ovine NKp46 followed by positive selection using pan-mouse IgG Dynabeads. NK cell purity was assessed by labelling cells with mouse anti-ovine NKp46 indirectly conjugated to goat anti-mouse IgG PE. Representative FACS plots illustrate the gating strategy used to identify bovine NK cells. Single cells were gated (A), followed by lymphocytes (B) which were negative for the live cell discriminator Sytox Blue (C). Additional file 2D indicates the purity of NKp46+ NK cells (pink histogram). Gates were set using unstained cells (blue histogram). Purities of positively selected NK cells were consistently >95%. [file 13567_2016_367_MOESM2_ESM.pptx]

## Slide 1
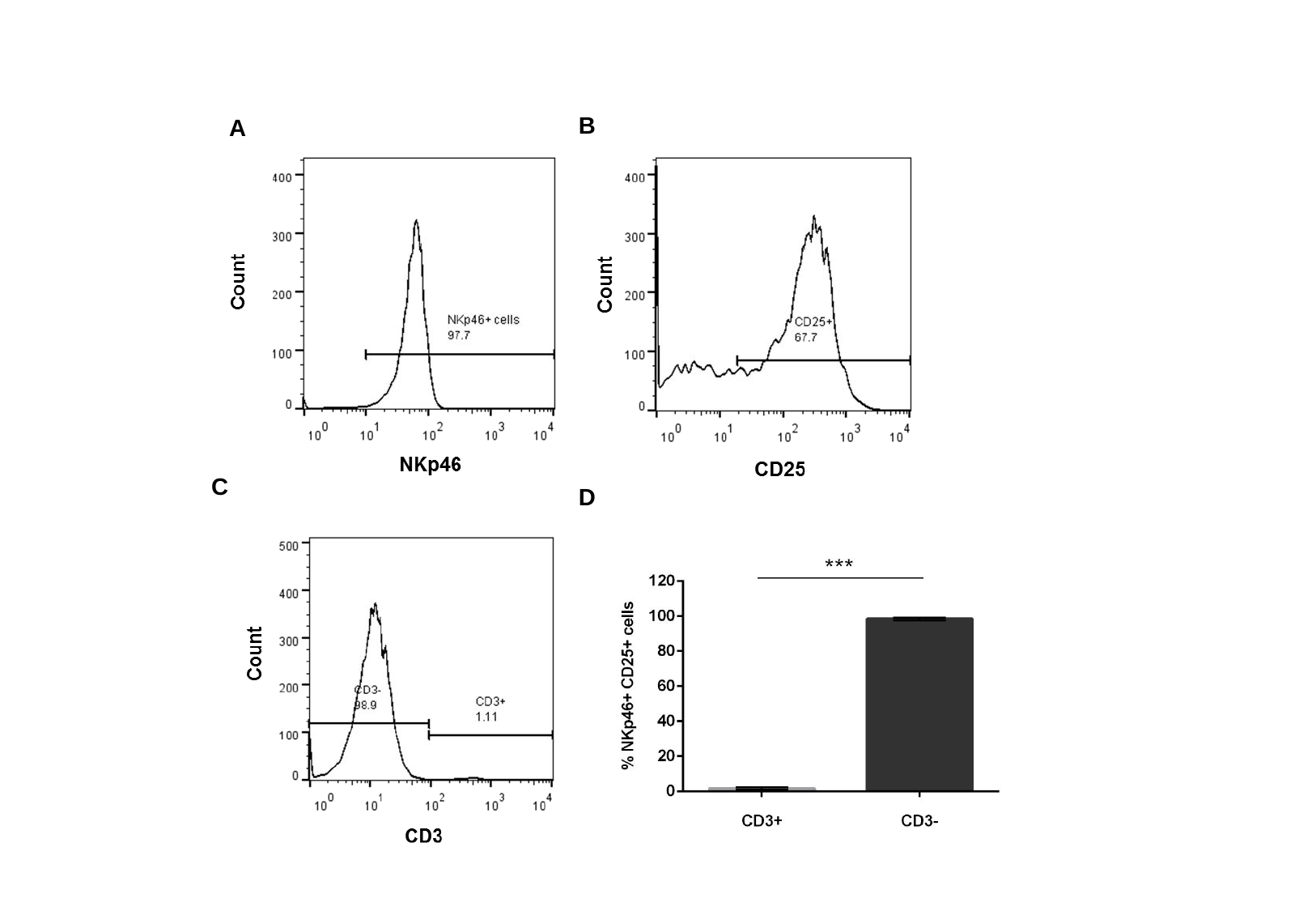

B
A
C
D

Supplement: Supplementary file 3 — 10.1186/s13567-016-0367-4 NKp46+ CD3+ lymphocytes were not activated following co-culture with BCG-infected DCs. Monocyte-derived DCs were cultured for three days and infected with BCG for 24 h. NKp46+ cells were enriched from peripheral blood and cultured with infected DCs at a ratio of 5 NK cells per DC. After 18 h of co-culture, cells were labelled with mAbs for CD3, NKp46 and CD25 and analysed by flow cytometry. FACS plots from one representative animal denotes the gating strategy used to assess the percentage of CD3+ and CD3- cells within the total gated NKp46+ CD25+ cells. Gates were set using FMO controls. NKp46+ NK cells were selected (A) followed by CD25+ NK cells (B) and then CD3+ and CD3- cells were gated (C). Pooled data from three calves illustrates the percentage of CD3+ (lighter bar) and CD3- (darker bar) cells ± SD within the NKp46+ CD25 population (D). Data were normally distributed (p > 0.05) and significance between CD3+ and CD3- cells was assessed using a 2-sample t-test; p < 0.001***. [file 13567_2016_367_MOESM3_ESM.pptx]
